# 

**Published:** 1889-09

**Authors:** 


### BOOK NOTICES.

THE ANNUAL OF THE UNIVERSAL MEDICAL SCIENCES FOR 1889 has jus t appeared. This valuable work is a complete report of the progress of the civilized world in medicine and surgery during the past year. It is edited by Prof. Chas. E. Sajous of Philadelphia, and the list of associate ediiors includes the most most prominent names in the medical fraternity of this country. There are besides some two hundred corresponding editors from all parts of the globe. With such a corps of writers, giving their actual personal experiences and experiments, the ANNUAL cannot fail to be one of the most important scientific contributions of its kind. It has many excellent explanatory illustrations and is issued in five volumes. In addition to being a resume of the doing of the doctors during the last twelve months, the ANNUAL is a review of the medical magazines for the same time. A new and useful feature is a thorough system of references to the various journals from which qnotations have been made. The ANNUAL has become not only a fixture in the scientific world but a necessity to every practising physician. Publishcd by F. A. Davis. Philadelphia, New York and London.

THE CENTURY, that primer of monthlys is becoming more and more a necessity in numberless households. Its wood cuts rival the most delicate steel engravings. Its excellence is admitted upon all hands. The Century Co. N. Y.

INEBRIETY, its Etiology, Pathology, treatment and Jurisprudence, Norman Kerr, M. D. L. H. S., second edition. Pubiished at London, England, by H. K. Lewis, 136 James St., W. C.

THE HOME MAKER. Published monthly at 29 West 22nd Street, New York City. The name of the editress of this magazine—Marion Harland—is a household word and would be sufficient reccommendation for a magazine eveu withont The Home Maker's attractive features.

